# Expression of hypoxia-inducible factor-1α, endothelin-1 and adrenomedullin in newborn rats with hypoxia-induced pulmonary hypertension

**DOI:** 10.3892/etm.2014.1728

**Published:** 2014-05-20

**Authors:** LE WANG, YING ZHOU, MINGXIA LI, YANPING ZHU

**Affiliations:** Department of Neonatology, The First Affiliated Hospital of Xinjiang Medical University, Ürümqi, Xinjiang 830011, P.R. China

**Keywords:** hypoxia-inducible factor-1, endothelin-1, adrenomedullin, pulmonary hypertension, rat

## Abstract

Hypoxia-inducible factor (HIF)-1α is associated with hypoxia-induced pulmonary hypertension (HPH) in adults. In the present study, the expression levels of HIF-1α*,* endothelin (ET)-1 and adrenomedullin (ADM) were analyzed during HPH in neonates. In total, 96 newborn rats were subjected to hypoxia or normoxia for 3, 5, 7, 10, 14 or 21 days (n=8 per subgroup). HIF-1α, ET-1 and ADM expression levels were measured by quantitative polymerase chain reaction. In addition, the intima-media thickness/external diameter ratio (MT%) and medial wall cross-sectional area/vessel total cross-sectional area ratio (MA%) were calculated to evaluate pulmonary vascular remodeling. The mean pulmonary arterial pressure (mPAP) increased with exposure to hypoxia. Furthermore, the expression levels of HIF-1α, ET-1 and ADM in the lungs were shown to increase after three and five days of hypoxia, while the MT% and MA% increased after seven days of hypoxia, as compared with the controls (P<0.05). Therefore, the expression of HIF-1α, ET-1 and ADM is upregulated in the lungs of newborn rats during early HPH. At later stages, the mPAP increases, vascular remodeling occurs and HIF-1α, ET-1 and ADM expression levels restore to normal levels.

## Introduction

Hypoxia-induced pulmonary hypertension (HPH) is a complication of numerous pulmonary conditions, which is associated with hypoxia. HPH is usually a life-threatening condition in neonatal populations. In conditions of severe hypoxemia (1), pulmonary vascular contraction and persistent cramp leads to persistent pulmonary hypertension with a poor prognosis. Unrelieved pulmonary hypertension can lead to right-sided heart failure and even mortality. However, the pathogenesis of neonatal HPH is not completely clear.

A number of adult studies (2–4) investigating the pathogenesis of HPH have been conducted in adult populations and animals. Hypoxia may lead to the injury of endothelial cells in the pulmonary arterioles, triggering the onset of pulmonary hypertension. Injury-induced dysfunction of the endothelium leads to the abnormal secretion of vasoactive substances and cytokines, thus, causes a functional imbalance between vasoconstrictors and vasodilators. This functional imbalance exerts effects on vascular smooth muscle cells, resulting in pulmonary vasoconstriction in the early stages, which is followed by pathological changes in the pulmonary vascular wall, vascular remodeling and pulmonary hypertension during the late stages. As a key nuclear transcription factor in hypoxic conditions, hypoxia-inducible factor (HIF)-1α is considered to be closely associated with the pathogenesis of HPH, functioning as the central link (5) in mediating pulmonary vasoconstriction and the remodeling of pulmonary vascular and hypoxia-induced gene expression. Endothelin (ET)-1 functions as an endogenous vasoconstrictor; with increasing mean pulmonary artery pressure (mPAP), the level of ET-1 (6–7) in the serum and lung tissue also increases. Adrenomedullin (ADM) functions as a vasodilator, thus, with increased levels of ADM (8), pulmonary vasodilation is gradually enhanced and the mPAP is reduced. ET-1 and ADM are key target genes of HIF-1α (9), which are involved in vasoconstriction, vascular remodeling and the development of HPH. Study into the pathogenesis of HPH is limited to adult and associated animal experiments, thus, research into neonatal HPH requires further investigation. It remains uncertain whether HIF-1α plays a similar role in the pathogenesis of HPH in newborns as it does in adults. The effects of HIF-1α, ET-1 and ADM in newborns with HPH are also unknown. Thus, the aim of the present study was to establish a model of HPH in newborn rats in order to evaluate the changes in the expression levels of HIF-1α, ET-1 and ADM and the effect of HIF-1α and its regulatory factors, ET-1 and ADM, in the blood serum and lung tissue. Providing information on the pathogenesis of HPH in newborns may aid the identification of novel treatments for HPH in neonates.

## Materials and methods

### Animal model

All the procedures involving animals were approved by the Animal Care Council and Animal Ethics Committee of the First Affiliated Hospital of Xinjiang Medical University (Ürümqi, China).

Healthy newborn Wistar rats (weight, 15–25 g; age, 3–5 days) were used in the study. In total, data were collected from 96 rats. Each animal was randomly assigned to one of the six subgroups in the hypoxic or control groups (n=8 per subgroup). In the hypoxic group, the subgroups were based on the duration of hypoxia (3, 5, 7, 10, 14 or 21 days), while the subgroups of the control group were subjected to normoxia for 3, 5, 7, 10, 14 or 21 days.

Rats in the hypoxic group were treated in order to create a model of HPH (10). All the rats in the hypoxic group were placed in a normobaric hypoxic chamber and a gas mixture of 8% O_2_ mixed with N_2_ was pumped into the chamber at a rate of 1.5 l/min. The fraction of inspired oxygen (FiO_2_) in the chamber was monitored using an oxygen analyzer (CY-100B; Lihua Science & Technology Co., Ltd., Hangzhou, China) and maintained in the range of 10±0.5%. Hypoxic conditions were sustained for 8 h per day (day/night ratio, 12/12 h). For each subgroup in the hypoxic group, the end point was at day 3, 5, 7, 10, 14 or 21 following exposure to hypoxia. The rats in the control group were maintained under similar conditions, but without being subjected to hypoxia.

### mPAP monitoring

At the predesignated time points (day 3, 5, 7, 10, 14 or 21), the rats from the appropriate control and hypoxic subgroups were anesthetized, placed on their backs, the trachea was exposed and punctured with a venous trocar and the trocar was then pushed into the trachea. An animal respirator (HX-200 small animal ventilator, Taimeng Technology Co., Ltd., Chengdu, China) was connected to provide mechanical ventilation with the following respirator parameters: Respiratory rate, 100–120 breaths/min and tidal volume, 2–3 ml/min). A longitudinal skin incision was made on the left sternal border, and blunt layer-by-layer separation of the tissues was performed until the heart was fully exposed. The tip of a scalp needle was punched through the base of the pulmonary artery and then orientated against the direction of the blood flow. The other end of the scalp needle was connected to a pressure sensor (BP-6; Thaimeng Technology Co., Chengdu, China) that measured the mPAP (11).

### Measurement of HIF-1α, ET-1 and ADM mRNA expression levels in the lung tissue

Lung tissue samples were collected from the rats within 5 min of sacrifice and stored in liquid nitrogen until required for analysis. Total RNA was extracted from 60–100-mg samples of the left upper lobe lung tissue. Quantitative polymerase chain reaction (PCR) was performed with each group. The RNA concentration and purity were determined using an ultraviolet spectrophotometer (SmartSpec Plus; Bio-Rad, Hercules, CA, USA), and cDNA was obtained using a reverse-transcription kit, according to the manufacturer’s instructions. The samples were stored at −20°C. The primers used for quantitative PCR analysis are shown in Table I and the β-actin gene was used as an internal reference to normalize the mRNA expression levels of HIF-1α, ET-1 and ADM. The conditions for quantitative PCR were as follows: 95°C for 30 sec; 40 cycles of 95°C for 10 sec, 53°C for 30 sec and 65°C for 10 sec; followed by a melting curve program.

### Measurement of vascular remodeling

Tissue samples were collected at sacrifice from the upper lobe of the right lung. The samples were fixed in 10% neutral formalin for one week, subjected to conventional paraffin embedding and 4-μm thick sections were prepared and stained with hematoxylin and eosin. Three sections were randomly selected from each rat, from which five pulmonary arterioles, next to the respiratory bronchioles and alveoli with relatively round cross sections and a diameter of 50–100 μm, were selected for analysis. The intima-media thickness/external diameter ratio (MT%) and medial wall cross-sectional area/vessel total cross-sectional area ratio (MA%) of the selected arterioles were analyzed using pathological image analysis software (PIPS-2020; Chongqing Tianhai Medical Equipment Co., Ltd., Chongqing, China) to evaluate the thickening of the pulmonary arteriole walls.

### Statistical analysis

Results are expressed as the mean ± standard error. One-way analysis of variance was used to evaluate the differences between two groups, where P<0.05 was considered to indicate a statistically significant difference. The analysis was performed using the SPSS version 18.0 software (SPSS, Inc., Chicago, IL, USA).

## Results

### Hypoxia increases the mPAP

Significant increases in the mPAP were observed in each subgroup of the hypoxic group when compared with the respective control subgroups of neonatal rats (P<0.05). The mPAP elevated as the duration of hypoxia increased in the hypoxic group (Table II).

### Hypoxia increases the mRNA expression levels of HIF-1α, ET-1 and ADM in the lungs

**Figure 1:**
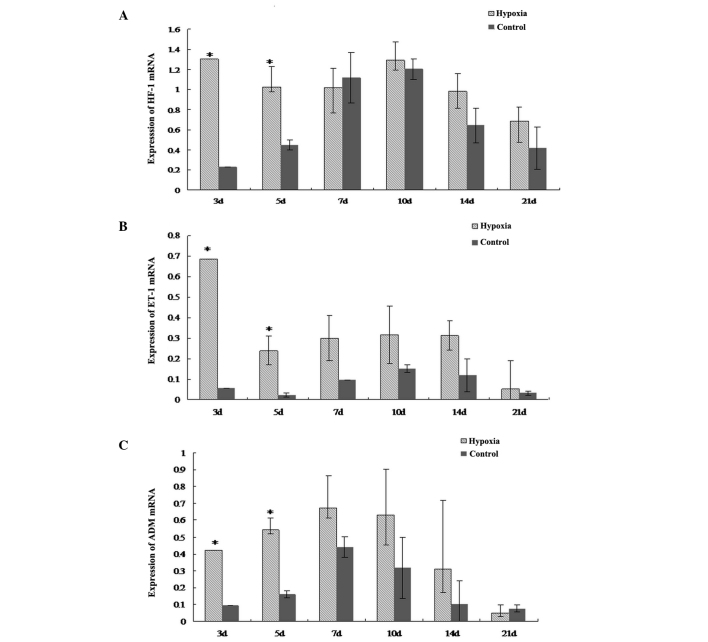
mRNA expression levels of (A) HIF-1α, (B) ET-1 and (C) ADM in the lung tissues from the various subgroups of newborn rats subjected to hypoxia or normoxia (n=8 per subgroup). Values are expressed as the mean ± standard deviation. ^*^P<0.01, vs. respective control subgroup. HIF, hypoxia-inducible factor; ET, endothelin, ADM, adrenomedullin.

The mRNA expression levels of HIF-1α, ET-1 and ADM in the lungs of the neonatal rats in the hypoxic group were higher at day three and five when compared with the respective control subgroups (P<0.05; Fig. 1). However, at the later time points, the mRNA expression levels of HIF-1α, ET-1 and ADM were not significantly different between the hypoxic and control groups.

### Pulmonary vascular remodeling

**Figure 2:**
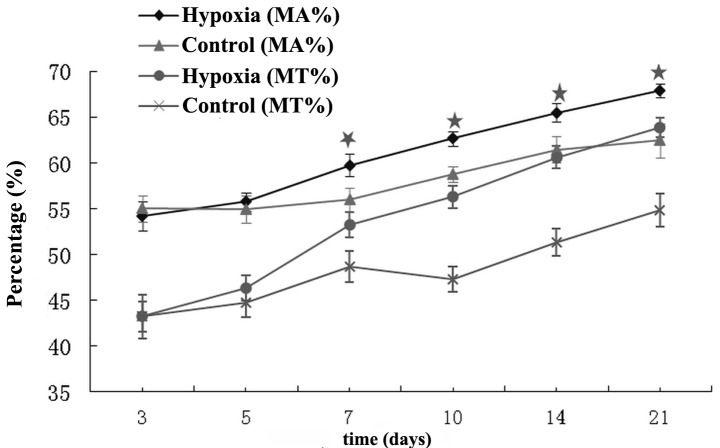
Effects of the various durations of hypoxia on the MT% and MA% ratios of the pulmonary arterioles in the various subgroups. ^*^P<0.05, vs. respective control subgroups. MT%, intima-media thickness/external diameter ratio; MA%, medial wall cross-sectional area/vessel total cross-sectional area ratio.

MT% and MA% ratios of the pulmonary arterioles increased in the hypoxic group from day seven onwards when compared with the respective control subgroups (P<0.05; Fig. 2).

## Discussion

Research into the pathogenesis of HPH has revealed that HIF-1α plays an important role in pulmonary vascular constriction, vascular remodeling (12–14) and the development of pulmonary hypertension, possibly by regulating the expression levels of ET-1 and ADM at a transcriptional level. HIF-1α expression is very sensitive to the intracellular oxygen concentration. Under normoxic conditions, HIF-1α is continuously synthesized and expressed in the cytoplasm, but is rapidly degraded by the ubiquitin-mediated pathway. However, under hypoxic conditions, HIF-1α expression exponentially increases and HIF-1α accumulates in the nucleus due to the inhibition of hydroxylation and protease degradation (15). The present study demonstrated that the mRNA expression levels of HIF-1α increased during the early stages of hypoxia, however, after seven days of hypoxia, HIF-1α mRNA was expressed at a similar level to the control group. This observation may be due to the development of tolerance to hypoxia. Under moderate hypoxic conditions, HIF-1α expression is predominantly regulated by changes in the oxygen concentration (2). In the present study, neonatal rats were continuously exposed to low concentrations of oxygen at a FiO_2_ of 10±0.5%, with no changes in the oxygen concentration. Therefore, at the late stages of hypoxia (day seven onwards), HIF-1α mRNA expression was not regulated by hypoxia and was similar to that of the control group. The results of the present study indicate that hypoxia induces high levels of HIF-1α mRNA expression in the lung tissues during the early stages of HPH development. However, the modulatory effects of hypoxia on HIF-1α expression become weaker during the late stages of hypoxia, with the HIF-1α expression levels reverting to normal levels, instead of increasing with the mPAP.

ET-1, a vasoconstrictive substance, is secreted by endothelial cells in response to injury or excessive activation (16). ET-1 exerts marked effects on vasoconstriction and vascular remodeling. The lung is one of the most important target organs for ET-1 metabolism (17). During the early stages of hypoxia, arterial endothelial cells are stimulated to secrete large amounts of HIF-1α, which further induces high expression levels of the HIF-1α target gene, ET-1. However, as there is a lack of dense granules in pulmonary endothelial cells for the storage of ET-1 mRNA, and ET-1 mRNA is unstable, ET-1 activity gradually decreases over time during hypoxia. In the present study, the mRNA expression of ET-1 increased during the first five days of hypoxia; however, no marked changes were observed after five days. During the late stages of hypoxia, severe damage to the endothelial cells leads to continually low levels of ET-1 secretion, and the degradation of ET-1 in the lung tissues also decreases. Therefore, ET-1 mRNA expression was not significantly different in the lung tissues of the hypoxic and control groups during the late stages of hypoxia. The results indicate that ET-1 may play an important role in the pathogenesis of HPH in newborn rats.

Imbalances between vasoconstrictors and vasodilators play a key role in the development of HPH. There are two HIF-1α-binding sites in the ADM promoter, and HIF-1α can regulate the transcription of ADM upon binding to the ADM promoter. Mutation of the HIF-1α-binding sites in the ADM promoter decreases the expression of ADM; therefore, HIF-1α regulates ADM at a transcriptional level. Numerous studies on adult rats have hypothesized that hypoxia can induce the synthesis of ADM (18, 19). In adult rats, ADM mRNA expression increases continuously in response to hypoxia, with the most pronounced increases observed in arterial endothelial cells and vascular smooth muscle cells. Increased ADM expression may be involved in lowering vascular tension (20) and alleviating hypoxia-induced damage to the lung tissues by inducing vasodilation. In the present study, HIF-1α and ADM mRNA expression levels significantly increased during the early stages of hypoxia when compared with the control group. This observation indicates that the accumulation of HIF-1α may upregulate ADM mRNA expression, which may enhance the synthesis and secretion of ADM in endothelial and smooth muscle cells. In response to prolonged hypoxia, vascular remodeling and endothelial injury occur and the activity of the endothelium-derived vasodilator pathway decreases, resulting in a gradual decrease in ADM synthesis and suppression of the effects of ADM on vascular smooth muscle cell proliferation (21). Finally, the potent vasoconstriction and cell proliferation promoted by ET-1 lead to an imbalance between vasoconstrictors and vasodilators, resulting in the development of vascular remodeling and pulmonary hypertension (22). These observations indicate that the vasoconstrictor, ET-1, dominates during the development of HPH in neonatal rats, whereas the vasodilator, ADM, exerts weaker effects.

In the present study, no marked pathological changes were observed in the pulmonary arterioles during the early stages of hypoxia. However, vascular remodeling occurred in the pulmonary arterioles after seven days of hypoxia. Therefore, the results indicate that seven days of hypoxia is the cut-off point at which the pulmonary arterioles undergo transition from functional change to anatomical change in pulmonary hypertension. Thus, seven days represents the appropriate period of time for early management to prevent pulmonary vascular remodeling during the progression of HPH. Further study investigating pulmonary vascular remodeling in newborns is required in order to provide accurate clinical guidelines for the treatment of HPH in neonates.

In conclusion, HIF-1α is an important factor in the development of HPH in newborn rats, and ET-1 and ADM are also involved in this process. The present study has confirmed that the mRNA expression levels of HIF-1α, ET-1 and ADM are elevated in the lung tissues during the early stages of hypoxia in newborn rats. However, during prolonged hypoxia, although the mPAP is elevated and vascular remodeling occurs in the pulmonary arterioles, the mRNA expression levels of HIF-1α, ET-1 and ADM are not significantly different when compared with control animals.

## Figures and Tables

**Table I:** PCR primers used in the study.

Gene	Primers	Amplified fragment length (bp)
β-actin	F: GGAGATTACTGCCCTGGCTCCTA R: GACTCATCGTACTCCTGCTTGCTG	150
HIF-1α	F: CAACTGCCACCACTGATGAAT R: CCACTGTATGCTGATGCCTTAG	133
ET-1	F: TGTTCAGACTGGCAGAGGAC R: CAAGAAGAGGCAAGAGAATCACT	120
ADM	F: GAGCGGACTGAGACAATC R: GTAAGTAATGAGGCGTATGC	192

PCR, polymerase chain reaction; F, forward; R, reverse; HIF, hypoxia-inducible factor; ET, endothelin; ADM, adrenomedullin.

**Table II:** Analysis of the mPAP in the various hypoxic and control subgroups (n=8 per subgroup).

Subgroup	mPAP (mmHg)
Day 3 hypoxia	8.59±1.57^b^
Day 3 control	6.14±1.02
Day 5 hypoxia	10.02±1.81^a^
Day 5 control	8.24±1.06
Day 7 hypoxia	11.63±2.56^b^
Day 7 control	8.33±0.76
Day 10 hypoxia	14.84±2.06^b^
Day 10 control	10.16±2.15
Day 14 hypoxia	15.29±2.88^b^
Day 14 control	10.92±2.74
Day 21 hypoxia	18.04±2.69^b^
Day 21 control	12.17±1.64

Values are expressed as the mean ± standard error.

a P<0.05 and

b P<0.01, vs. respective control subgroup.

mPAP, mean pulmonary arterial pressure.
